# CD123 target validation and preclinical evaluation of ADCC activity of anti-CD123 antibody CSL362 in combination with NKs from AML patients in remission

**DOI:** 10.1038/bcj.2017.52

**Published:** 2017-06-02

**Authors:** L H Xie, M Biondo, S J Busfield, A Arruda, X Yang, G Vairo, M D Minden

**Affiliations:** 1 Department of Medical Oncology and Hematology, Princess Margaret Cancer Centre, University Health Network, University of Toronto, Toronto, Ontario, Canada; 2 Department of Hematology, Huashan Hospital, Fudan University, Shanghai, China; 3 CSL Limited, Bio21 Institute, Parkville, Victoria, Australia; 4 Department of Medicine, University of Toronto, Toronto, Ontario, Canada

## Abstract

Despite the heterogeneity of acute myeloid leukemia (AML), overexpression of the interleukin-3 receptor-α (CD123) on both the more differentiated leukemic blast and leukemic stem cells (LSCs) provides a therapeutic target for antibody treatment. Here we present data on the potential clinical activity of the monoclonal antibody CSL362, which binds to CD123 with high affinity. We first validated the expression of CD123 by 100% (52/52) of patient samples and the correlation of NPM1 and FLT3-ITD mutations with the high frequency of CD123 in AML. *In vitro* studies demonstrated that CSL362 potently induced antibody-dependent cell cytotoxicity (ADCC) of AML blasts including CD34^+^CD38^−^CD123^+^ LSCs by natural killer cells (NKs). Importantly, compared with healthy donor (HD) NKs, NKs drawn from AML patients in remission had a comparable ADCC activity against leukemic cells; of note, during remission, immature NKs were five times higher in AML patients than that in HDs. Significantly, we report a case where leukemic cells were resistant to autologous ADCC; however, the blasts were effectively lysed by CSL362 together with donor-derived NKs after allogeneic hematopoietic stem cell transplantation. These studies highlight CSL362 as a promising therapeutic option following chemotherapy and transplant so as to improve the outcome of AML patients.

## Introduction

In the past 40 years, the standard treatment of acute myeloid leukemia (AML) has relied on cytotoxic chemotherapy combinations; despite changes in the drugs being used, there has been little improvement in outcomes. With current induction chemotherapy regimens complete remission (CR) occurs in up to 80% of patients;^1, 2^ however, even with several cycles of consolidation chemotherapy most individuals experience a relapse and die of their disease. Allogeneic hematopoietic stem cell transplantation (allo-HSCT) is considered definitive consolidation therapy in first or later CR, albeit at the expense of high morbidity and mortality due to infections and graft versus host disease;^3, 4^ the predominant benefit of allo-HSCT is considered to be the effect of the immune-mediated graft versus leukemia that occurs post transplant. Novel therapies with increased efficacy as compared with chemotherapy alone and decreased toxicity associated with allo-HSCT are clearly needed for the treatment of AML.

In the last 15 years, an increasing body of evidence has shown that engineered antibodies can improve the outcome of patients with cancer, including AML.^5^ Among the various potential antigen targets for antibody-based AML therapy, CD123 (interleukin-3 receptor-α, an important growth and differentiation receptor for early hematopoietic cells and the myeloid lineage) has attracted much attention because of its high expression in AML and in particular on leukemic stem cell (LSCs).^6, 7, 8^ In general, LSCs have an inherent resistance to traditional chemotherapeutics associated with their stem cell nature; their persistence following chemotherapy is responsible for disease relapse.^9, 10, 11, 12^ A number of different approaches for targeting CD123 are being investigated, either using its toxin-modified natural ligand, small molecules to inhibit receptor signaling or specific monoclonal antibody.^13, 14, 15^ It has previously been reported that antibodies against CD123 can mediate cytolysis of AML cells and in particular of LSCs.^16, 17^ Although active, these antibodies were of murine origin and lacked the ability to efficiently recruit highly active human effector cells. More recently, a second-generation anti-CD123 antibody (CSL362) has been developed. CSL362 was engineered towards human IgG (humanized) and to have increased affinity to the Fc receptor CD16 on natural killer (NK) cells. This antibody has been shown to effectively induce antibody-dependent cell-mediated cytotoxicity (ADCC) of AML blasts *in vitro* and in AML xenograft models.^18^ Of interest was our observation that autologous NK cells from a patient with chronic myeloid leukemia blast crisis-induced CSL362-mediated cytotoxicity to the same extent as allogeneic healthy donor (HD) NK cells.^19^

NK cells provide a natural immunity against cancer. In AML, a role for NK cells in controlling disease is supported by the association of improved outcome of patients who have high levels of NK cells following allogeneic stem cell transplant.^20, 21^ Of note, it has been found that NK cells developing post chemotherapy and post transplant can have an immature phenotype; it has been suggested that these immature post-transplant NK cells have a reduced ability to kill recipient blast cells.^22, 23, 24^ In the present study we found that post chemotherapy autologous NK cells and NK cells from HDs produced comparable lysis of AML blast cells treated with CSL362. The results strongly encourage the clinical development of CSL362 as a novel therapeutic antibody for AML patients.

## Materials and Methods

### Patients and samples

Peripheral blood (PB) samples of all AML patients and HDs were obtained following informed consent as approved by the research ethics board of the University Health Network. PB mononuclear cells (PBMCs) were separated by Ficoll (GE Healthcare, Uppsala, Sweden) density gradient centrifugation. Fifty-two patients (median age 56 years; range 23–86) with newly diagnosed AML treated by the Division of Medical Oncology and Hematology, Princess Margaret Cancer Center between April 2007 and January 2013 were enrolled in the study for assessing CD123 expression. The diagnosis of AML was made by expert clinical hematopathologists according to the morphological and immunological criteria of the National Cancer Institute expert panel and World Health Organization guidelines. Twenty patients in the first CR were enroled in ADCC-mediated blast depletion assays. The characteristics of these patients are shown in Table 1. Most patients received daunorubicin 60 mg/m^2^ for 3 days and cytarabine 100 mg/m^2^ (age ⩾60 years) or 200 mg/m^2^ (age <60 years) by continuous infusion daily for 7 days. CR was defined as the presence of normal erythropoiesis, granulopoiesis and megakaryopoiesis, and <5% leukemic blasts in the bone marrow, and PB absolute neutrophil count ⩾1 × 10^9^/l and platelet count ⩾100 × 10^9^/l. PB samples from remission patients were collected at varying time points after being in the first CR for at least 1 month (see Table 1).

### CD123 expression analysis

The expression of CD123 was analyzed by flow cytometry (BD FACSCanto II) using PE-conjugated anti-CD123 antibody (Clone 9F5, BD Biosciences, Franklin Lakes, NJ, USA). To determine CD123 expression on leukemic bulk and subsets of blast, CD45-FITC (2D1^5^), CD34-APC (581), CD33-v450 (WM53) and CD38-PE-Cy7 (HB7) were co-stained with CD123-PE. All antibodies were purchased from BD Biosciences. A CD45dim/SSClow gate was used to limit the analysis of AML bulk cells.^25^ Geometric mean fluorescence intensity (MFI) values of CD123 measured using FlowJo (Tree Star Inc., Ashland, OR, USA) were normalized to the MFI of the residual normal lymphocytes within each sample, which were negative for CD123. Total 52 samples’ MFI ratios (MFI blast/MFI lymphocyte) were subsequently analysed for CD123 expression intensity. Samples with CD123MFI ratio ⩾2.0 were regarded as positive.

### Analysis and enrichment of NK cells

PBMCs were stained with monoclonal antibody combinations of CD3-PE-cy7, CD56-FITC and CD16-PE (IgG1, BD Biosciences) to characterize the NK cell phenotype and CD16^+^ expression on NK cells. NK cells were then enriched from PBMC using negative selection kits from Miltenyi Biotec (Bergisch Gladbach, Germany). The enriched NK cells were resuspended in RPMI medium containing 10% fetal bovine serum for immediate use in ADCC assay. Before the use for ADCC, an aliquot of these enriched NK cells (CD3^−^/CD56^+^) were further characterized for purity and the proportion of mature NK cells (CD56low) and immature NK cells (CD56high) by flow cytometry.

### CSL362 antibodies

CSL362 and the corresponding IgG1 isotype control antibody BM4 were produced by CSL Limited, Parkville, Australia. CSL362 was developed from the murine anti-CD123 monoclonal antibody 7G3 in a stepwise process of humanization, affinity maturation and Fc-engineering. Two amino acid mutations (S239D and I332E) were introduced into the Fc region to enhance NK-mediated cytotoxicity of the antibody. The characterization of CSL362 activity has been reported in previous studies.^17, 18, 26^

### *In vitro* AML blast depletion assay

In this study we refer to the cell-mediated blast depletion assay with CSL362 as ‘ADCC’, which was tested as follows: AML samples obtained at diagnosis were pretreated with CSL362 at 10 μg/ml (30 min) and incubated with enriched NK cells at an effector to target cell ratio (E:T) of 10:1 for 24 h in RPMI/10% fetal calf serum at 37 °C; residual AML cells were analyzed by flow cytometry. The CD45dim/SSClow population was used as the standard gate for AML blast cells. In some cases it was difficult to analyze the blasts, as the blast population was very close to the CD45highCD123^−^ population. For such samples, we used a combination of markers such as CD33, CD34 and CD38 to help clearly identify the leukemic blast cells. Apart from control cultures incubated with no antibody, pretreating the blast with BM4 was the nonspecific antibody control. The specific lysis mediated by CSL362 was calculated by the formula: remaining cells (%) of BM4−remaining cells (%) of CSL362)/remaining cells (%) of BM4.

### Statistical analyses

Graphpad Prism 6.0 (GraphPad Software, San Diego, CA, USA) was used for statistical analyses and graphs. Differences between two groups were compared using an unpaired *t*-test. Differences between three or more groups were compared using a one-way analysis of variance. For all analyses, mean±s.e.m. values are given, and *P<0.05 and **P<0.01, unless otherwise stated.

## Results

### CD123 is a widely expressed target in AML

**Figure 1:**
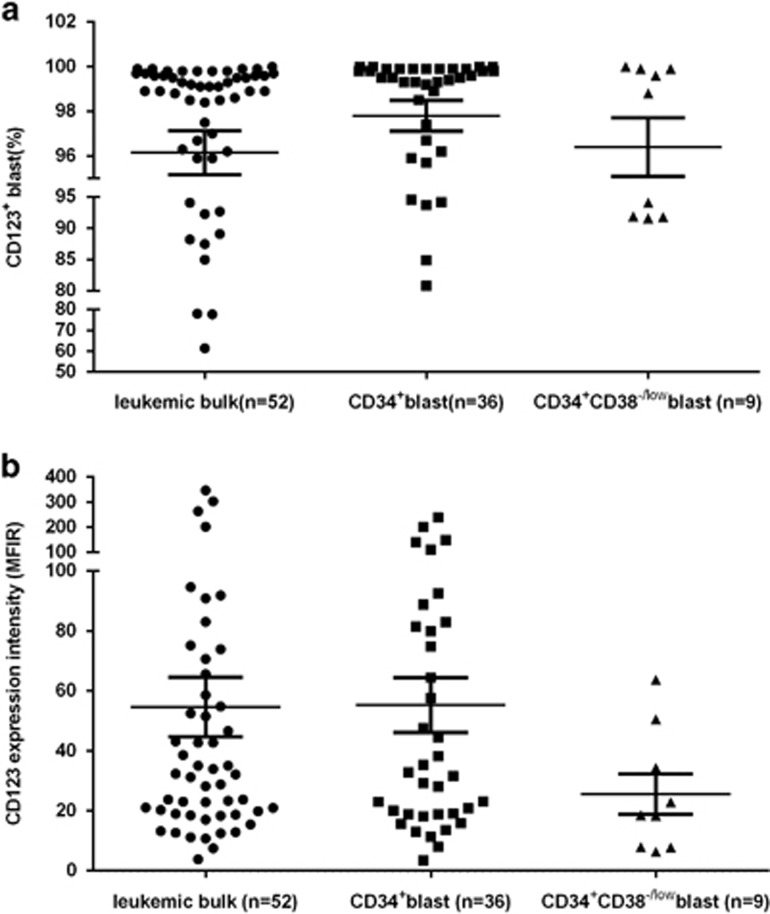
CD123 expression in AMLs. Fifty-two of 52 AML samples tested positive for CD123 with no difference in expression between leukemic bulk and leukemic progenitor cells. Leukemic bulk (blast: circles) population was gated by using standard side scatter (SSC) low CD45dim. CD123 expression on leukemic bulk was compared to that on CD34^+^ blast (squares) and that on CD34^+^CD38^−/^low LSCs (triangles), as well in the entity of all AML samples for frequency (**a**) and for intensity (**b**). Although all samples were scored as positive, the majority of samples had a relatively low-intensity staining for CD123.

In order to select appropriate AML patients for future CSL362 therapy, the frequency and intensity of CD123 expression was evaluated for 52 PBMC samples of newly diagnosed AML patients. CD123 was expressed by all AML blasts at variable levels (Figure 1). CD34^+^ blast cells were observed in 69.23% (36/52) of AML samples and all expressed CD123. A CD34^+^CD38^−/^low blast subset typically containing LSCs^27^ was observed in 17.31% (9/52) of the tested AML samples and all expressed CD123. No statistical difference was observed between the subsets in either expression frequency or expression intensity of CD123 in all AML samples (Figures 1a and b).

### CD123 expression correlates with NPM1 and FLT3-ITD status

**Figure 2:**
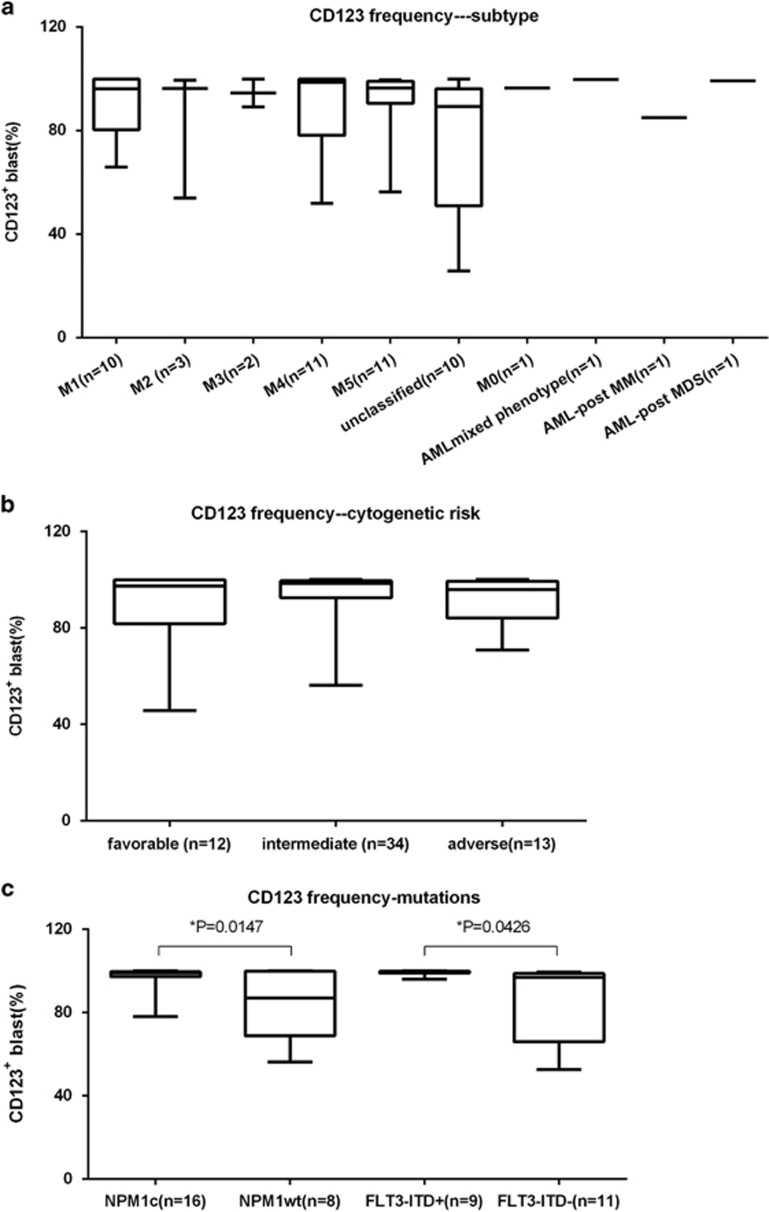
CD123 expression correlated with disease characteristics. (**a**) No difference was seen between AML subtypes. (**b**) No difference was seen between cytogenetic risk groups. (**c**) CD123 expression was statistically higher in samples with either NPM1 mutation or FLT3-ITD mutation than without those mutations.

Expression of CD123 by disease characteristics was assessed. There were five samples each diagnosed with AML-M0, AML-mixed, AML-post MPD, AML-post MM and AML-post MDS, respectively. The other enroled patients were classified into six FAB/WHO subtypes of AML: M1-M5 and Unclassified. CD123-positive cells were present in all subtypes; however, there was variability with regard to the proportion of CD123-positive cells (Figure 2a). By cytogenetic risk classification, no significant differences were seen between the three groups with regard to CD123 expression (Figure 2b). Consistent with previous data,^28^ the frequency of CD123expression was statistically higher in samples with a NPM1 mutation irrespective of FLT3-ITD status and vice versa (Figure 2c).

### NK cell numbers and phenotype in AML remission samples

**Figure 3:**
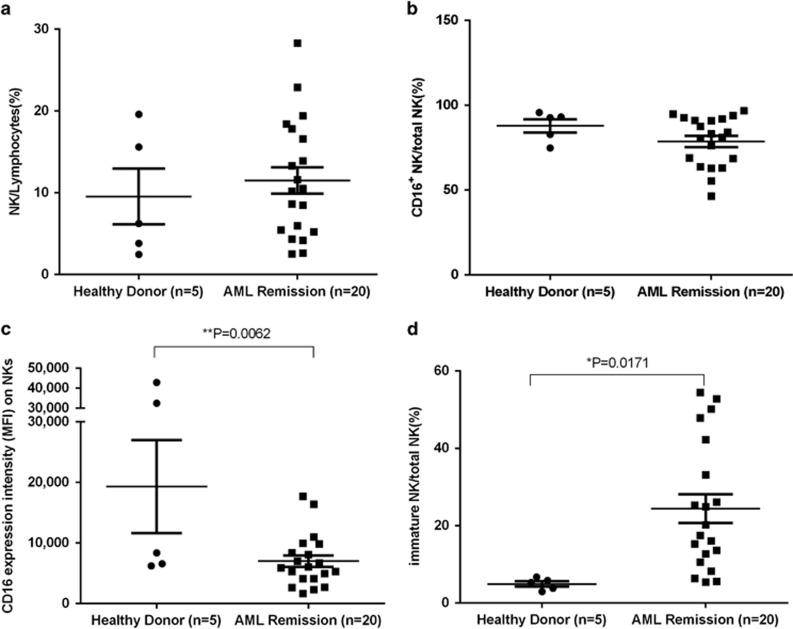
Profile of recovery NK cells in remission AML. PB was drawn from 5 HDs and 20 AML remission patients for NK phenotype analysis by flow cytometry. (**a**) Compared with HDs (circles), NK cells accounted for the similar percentage of lymphocytes in the PB of AML patients in remission (squares). (**b**) The CD16^+^ NK cells (ADCC functional NK cells) of patients were also comparable in frequency to that of HDs. (**c**) However, the intensity of CD16 expression on NK cells in remission AML was statistically lower than that in HD. (**d**) The percentage of CD56bright immature NK cells was higher in remission patients than that in HDs.

The NK cell population was assessed in AML patients at varying time points during the first CR. In spite of the higher median value and wider range, the proportion of CD3^−^CD56^+^ NK cells within the total lymphocyte population in AML patients was statistically similar to that found in HDs (Figure 3a). Furthermore, the percentage of CD16^+^ NK cells did not differ between HDs and AML patients (Figure 3b). However, the expression intensity of CD16 on patients’ NKs was significantly lower than that on HDs’ NKs (Figure 3c). When classifying NKs into CD56bright and CD56dim subpopulations, 25.88%±4.37% of the patients’ MACS-enriched NKs were CD56brightCD16^−/^low, characteristic of immature NKs. This was statistically higher than 4.49%±0.81% observed in HDs (*P*=0.0171) (Figure 3d). These findings indicate that NK cells return to normal levels after chemotherapy, but show a greater proportion of CD56bright immature NK cells and the corresponding lower intensity of CD16 expression at least over the time frame analyzed here.

### Autologous remission NK cells are potent effectors of CSL362-mediated ADCC against primary AML blast cells

**Figure 4:**
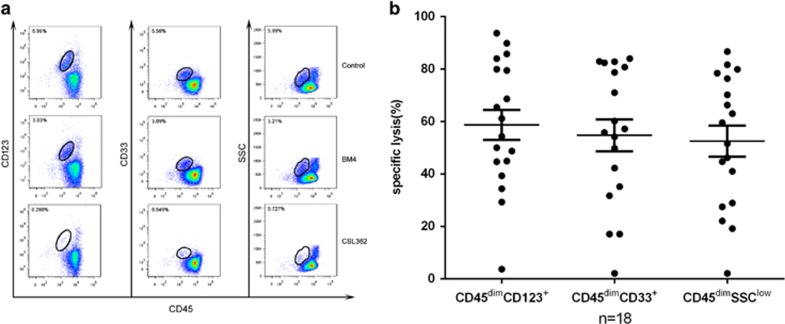
Autologous ADCC assays. (**a**) NK-mediated lysis of cells from Pt.130429 was assessed in cells treated with CSL362 or the control antibody BM4. There was an ~80% reduction in blasts in the CSL362 as compared with BM4-treated population. (**b**) No blast depletion was observed in 2of 20 samples. To summarize the data from 18 samples, the degree of killing with autologous NKs ranged from 30–90%, with no statistical difference between the three gating strategies.

To evaluate the ability of NK cells from AML patients in remission to kill autologous CD123-expressing blast cells, we established a flow cytometry-based ADCC assay using viably cryopreserved blasts obtained at the time of presentation as targets; autologous NK cells collected at different time points in remission, post consolidation, were used as effectors. In this assay, a CD45dim/SSClow gate was used to identify the leukemic blast cells. In addition, two other gates were used to confirm the leukemic blasts: CD45dim/CD33^+^ and CD45 dim/CD123^+^, respectively. The utility of this approach to identify consistent depletion of CSL362-treated leukemic blasts is shown for Pt.130429 (Figure 4a); similar results were found for 18 other patients. Of the 20 patients tested, only 2 cases (Pt. 130363 and Pt.130897) showed little depletion of CSL362-treated leukemic blasts in the presence of autologous, post chemotherapy NK cells. For Pt.130897 this may have been due to the low number of NK cells available in the remission blood sample; for this patient the PBMCs were collected on d120 post remission (Table 1) when the white blood cell count was 0.7 × 10^9^/l and lymphocyte count was 0.3 × 10^9^/l. Although the NKs percentage of lymphocytes had recovered to normal levels, the absolute number of NK cells was still lower than normal. For the 18 patients demonstrating NK-mediated ADCC, the mean specific lysis was >50% (Figure 4b). Intriguingly, there was no correlation between the frequency and intensity of CD123 expression on AML blasts and lysis. These data support the premise that AML leukemic blasts are susceptible to ADCC lysis in the presence of post recovery NK cells and CSL362.

### Allogeneic donor NK cells induced CSL362-mediated ADCC to the same level as autologous NK cells

**Figure 5:**
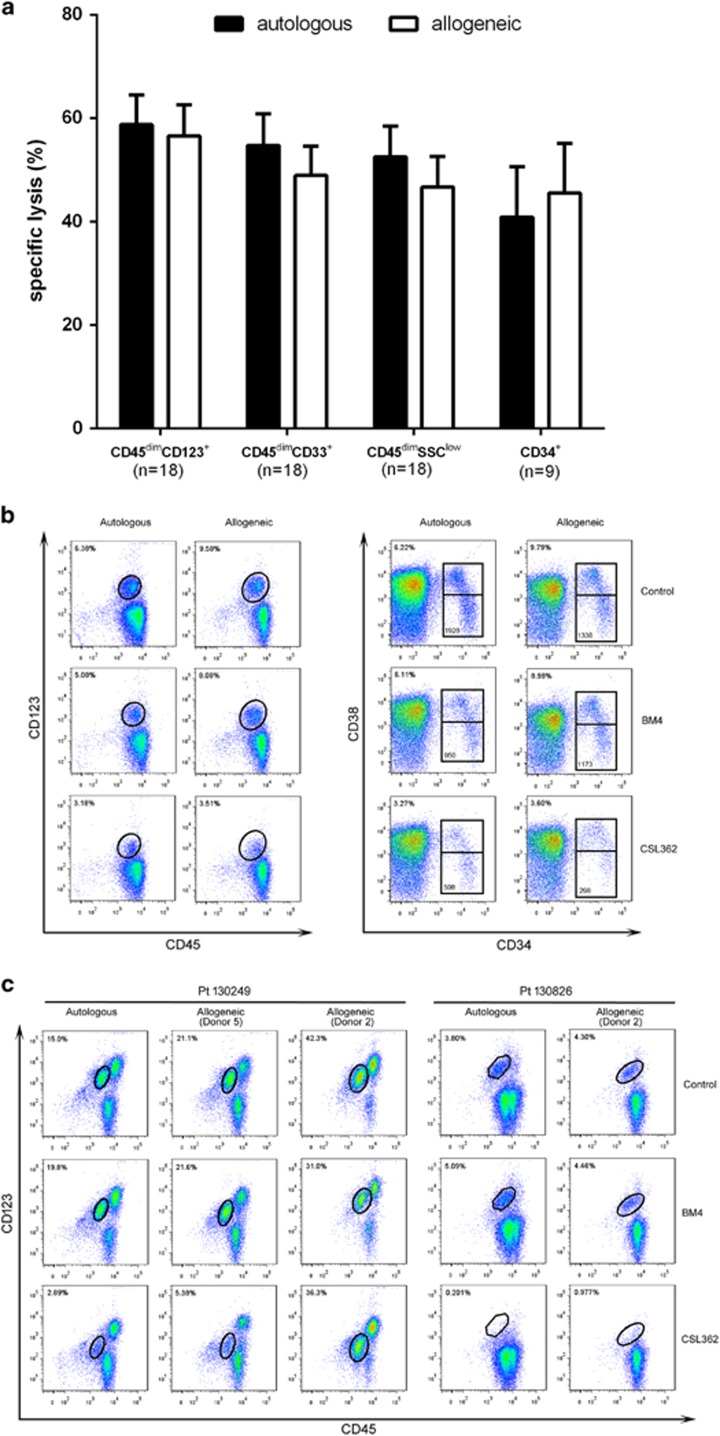
Autologous ADCC vs allogeneic ADCC. Allogeneic- and autologous-ADCC assays were performed for each sample at the same time and under the same conditions. (**a**) Autologous ADCC led to the specific lysis of blasts to the same level as allogeneic ADCC measured using different gating strategies on flow cytometry. (**b**) Representative data from Pt.130302 shows the autologous ADCC is comparable to allogeneic ADCC, through either of which CSL362 induced the depletion of not only AML blast (represented by the percentage on CD45 vs CD123 plots) and CD34+ blast (represented by the percentage on CD34 vs CD38 plots), but also LSC-enriched population (represented by the number of events occurring in the CD34^+^CD38^−/^low gate). (**c**) Differential killing by donor cells was observed. For pt 130249 a high degree of killing was observed with autologous (85.4%) or donor 5 NKs (75.1%). Although donor 2’s cells did not kill 130249 cells, they did effectively kill blasts from pt 130286.

As there was variable killing of AML cells by the combination of autologous NK cells and CSL362, we were interested in determining whether this was due to a defect in the patients’ own NK cells or an intrinsic resistance of the leukemic blast cells. NK cells were isolated from HDs and tested for their ability to kill leukemic blast cells in the presence or absence of CSL362 as described above. Figure 5a summarizes patients’ data confirming the observation that allogeneic-derived NKs can induce CSL362-mediated ADCC to a similar degree as observed with autologous NK cells. This confirms that AML blast cells are sensitive to NK cell-mediated ADCC in the presence of CSL362, regardless of the origin of the NK cells. Figure 5b represents data for patient 130302, highlighting the efficacy of CSL362 using either autologous or allogenic NK cells. The specific lysis of CD45dim/CD123^+^ blast population with CSL362 treatment was 37.4% (autologous) and 56.56% (allogeneic). Focusing on the CD34+ subset, the net effect of treatment with CSL362 was a specific lysis of the CD34^+^ cells by 46.48% (autologous) and 59.91% (allogeneic) relative to the control BM4 antibody. Furthermore, there was a significant killing of cells in the LSC population defined by CD34+CD38^−/^low CD123+. Of note, there was equivalent killing of the CD34+CD38^−/^low and the CD34+CD38+ population of blast cells.

**Figure 6:**
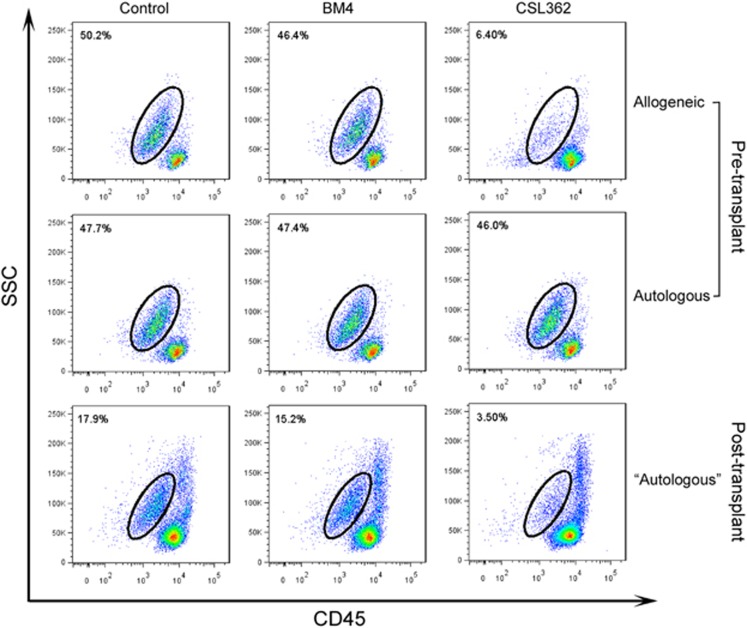
CSL 362-mediated killing of Pt 130363 cells pre and post allo-HSCT. Pre allo-HSCT, CSL362 induced specific lysis of blast with normal NKs from HD (top row), whereas no lysis with recovery NKs from remission patient (middle row). Post allo-HSCT, CSL362-induced specific lysis again with patient’s ‘autologous’ NKs, which are derived from the donor of allo-HSCT (bottom row).

Although in most cases we observed roughly the same degree of killing by autologous and allogeneic NK (allo-NK) cells, there were significant exceptions. First, although autologous killing tended to be high across samples, there was one case (Pt 130363) in which no killing was observed when autologous NK cells were used. However, the same AML blasts were specifically killed by allo-NK cells (see Figure 6). Second, there appeared to be selectivity in killing by allogeneic donor cells. This was evident as cells from the same HD showed variable degrees of killing of AML blasts from different patients; this was seen with the NK cells from one of the five donors tested. Conversely, AML cells from the same patient showed differential killing by cells from different donors. This was most dramatically seen for sample 130249 where allo-NK cells from donor 5 produced 75.09% killing, whereas the allo-NK cells from donor 2 produced no killing. This was not due to a general defect in donor 2 NK cells, as these cells efficiently killed the AML blasts of other cases (Figure 5c). Finally, we observed in 2/20 cases that cells outside the blast gate, which were CD123 positive, were not killed by the combination of anti-CD123 and NK cells (data not shown).

### CSL362 is efficacious in the setting of allo-HSCT for AML

The above variability in the lysis of target cells is highlighted by one case in which we had the opportunity to study NK cells from the patient pre and post allogeneic stem cell transplant. The leukemic blast cells (130363) were resistant to killing by autologous NK cells collected pretransplant. However, donor-derived NK cells obtained from the patient post-transplant were highly efficient in killing the leukemic cells. Similarly, NK cells from a HD also killed 130363 blast cells (Figure 6).

## Discussion

Treatment options after induction of remission for AML generally consist of intensive consolidation chemotherapy followed by a watch and wait period or allo-HSCT. Although transplant reduces the occurrence of relapse, patients may die due to complications or have a reduced quality of life due to the effects of graft versus host disease. Unfortunately, a large proportion of patients who do not undergo a transplant due to lack of a suitable donor, comorbidities or age not compatible with transplant, or patient choice continue to have a high rate of relapse. Hence, there remains a need for safe, tolerable and effective therapy to maintain/consolidate AML remission, especially for patients at high risk of relapse.^29^ A desired agent should effectively and specifically target both bulk AML cells and in particular the residual LSCs that are thought to be responsible for relapse and poor disease outcome.^30, 31^

In recent years, numerous reports have documented the presence of high interleukin-3 receptor-α (CD123) expression on the surface of AML blasts and the AML-LSC fraction, but not on normal HSCs. This identifies CD123 as an attractive target for antibody-based immunotherapy.^6, 16^ Indeed, our present results are in line with those data regarding the high expression of CD123 on bulk AML cells and the CD34^+^CD38^−^ fraction, which typically contains LSCs. As one of the aims of this study was to determine the degree of CSL362-enhanced NK cell killing of AML cells as it relates to the cell surface expression of CD123, we focused on the mean expression value/intensity as well as the proportion of cells above a certain threshold. In keeping with other studies,^13, 28^ and in contrast to a report by Jordan *et al.*,^6, 32^ we did not observe that CD123 expression was higher on the LSC containing CD34^+^CD38− blasts than bulk leukemia cells. The variable findings between groups might be explained by different type of samples tested (bone marrow or PB), different methods of expression investigation (flow cytometry or immunohistochemistry), different equipment, different gating strategies, different reagents and different size of cohorts evaluated in the studies. Regardless, all studies are in agreement that CD123 is expressed on cells within the stem cell compartment of the leukemic population and hence is a desirable therapeutic target. We further correlated CD123 expression with four disease characteristics. In accordance with a previous report,^6^ we observed that CD123 is overexpressed by most AML cells irrespective of subtypes investigated in the study, suggesting that targeting this molecule could be of wide benefit. Our data also confirmed a recent study showing no correlation between CD123 expression and risk groups.^28^ Likewise, that same study reported the correlation of higher levels of CD123 expression with NPM1 and FLT3-ITD mutations, which is consistent with our findings and other studies^33, 34^ Consequently, any study targeting CD123 should be sure to ensure inclusion of such patients and be powered to assess this group separately.

Previous studies have demonstrated that NK cells are an important mediator of AML cell killing, particularly in the post stem cell transplant setting.^35, 36, 37^ Following on the demonstrated importance of NK cytotoxicity, CSL362 was developed as a second-generation anti-CD123 antibody, which has over 100-fold increased affinity for CD16 on NK cells through its modified Fc domain. Indeed, our previous evaluation of neutrophils, monocytes, macrophages and NK cells in ADCC assays revealed that NK’s are the major effector cell responsible for CSL362-mediated cytotoxicity.^17^ Moreover, binding of CSL362 to NK cells leads to evidence of NK cell activation as shown by the upregulation of the degranulation marker CD107a, a prerequisite for NK cytolysis.^17^ In the current study, CSL362 or BM4 was added to the bulk blast cell population, without the addition of excess NK cells. Although there was blast depletion with CSL362, no killing was observed with the control BM4 antibody. This indicates that CSL362 can mediate its function through autologous NK cells in the presentation sample (data not shown). In AML, the functional NK cell compartment is decreased at initial presentation;^38, 39^ however, in remission numerous reports have documented that by four weeks post treatment NK cells show significant recovery, being among the earliest lymphocytes to come back.^40, 41^ In keeping with this, we observed that NK cells isolated from PB drawn 1 month or more after achieving the first CR had recovered to normal proportions. Combined, these findings help to dispel the concern that too few NK cells may be present in remission patients for CSL362 to be effective. Meanwhile, our studies indicate that the recovered NK cell population post chemotherapy contains a higher proportion of immature NK cells. This has been observed previously,^22^ where it was suggested that these cells might be deficient in cytolytic activity since immature NK cells are reported to be capable of cytokine and chemokine production but low killing ability; this is in contrast to mature NK cells that are considered to have robust cytotoxicity.^42^ However, the studies presented here indicate that NK cells developing post chemotherapy in AML patients are equal to HDs’ NKs in terms of cytolytic activity via CSL362-meidated ADCC despite the high frequency of CD56brightCD16^−^low immature NKs. Given the heterogeneity of AML, we believed it to be essential to test CSL362 against more than a few primary AML samples. Here we tested a collection of 20 samples that included six common subtypes of AML. Our data demonstrate that PB NK cells of AML patients post chemotherapy are capable of killing autologous blast cells in the presence of CSL362 across a range of AML subtypes.

The observation that NK cells could kill autologous AML blasts treated with CSL362 in the presence of a large number of blast provides encouragement that NK cells will be able to eliminate CSL362 binding AML cells *in vivo* when there is a low level of residual disease during the remission state. We are further encouraged by our finding that CSL362-mediated specific lysis of the CD34^+^CD38^−^CD123^+^ subset of AML blasts, which in general, more precisely, defines the CD34^+^ cellular component that contains LSCs.^43^ This is of importance, as these LSCs are believed to be responsible for disease relapse^30, 44^ and therefore elimination of these cells is crucial for reducing the chance of disease recurrence. As the residual LSCs have proven themselves to be resistant to standard chemotherapy, probably due to their reduced cycling and residence within the protective bone marrow niche, an alternative form of cell killing is required in order that they be eliminated; killing by CSL362 in conjunction with NK cells provides such an opportunity.

Our results are particularly striking when one compares ADCC mediated by CSL362, and autologous and allo-NK cells. The finding that for 18/20 samples autologous NKs obtained during remission induced ADCC that was comparable to HD allogeneic ADCC suggests that most AML patients may gain clinical benefit from the administration of CSL362 during remission by prolonging the time to relapse or even preventing relapse. For those patients whose autologous NK cells did not kill CSL362-treated blasts, allogeneic cells were effective. Already, haploidentical NK cells are being used in clinical trials to reduce relapse of AML.^45, 46^ Our studies suggest that the inclusion of the lineage-specific antibody CSL362 could further enhance the effectiveness of this approach for all patients. In addition to the use of CSL362 for the above adoptive immune-therapy, our finding that blasts of Pt.130363 were resistant to killing by autologous NK cells, but were sensitive to donor NK cells derived from the patient post allo-HSCT suggests that CSL362 could be used in the post-transplant setting to potentially enhance the therapeutic benefit of the transplant, particularly in settings where the post transplant relapse rate is high. The addition of CSL362 post allogeneic transplant may also allow for different anti-GvHD approaches allowing for reduced morbidity of this complication. These and other results provide the rationale for testing the tolerability and potential clinical efficacy of CSL362 in AML. It is currently being tested in a Phase I study of CSL326 in patients with CD123^+^ AML in remission (NCT01632852, Clinical Trials Gov).

## Figures and Tables

**Table 1:** Clinical information of 20 patients enroled for ADCC assay

*Patient no.*	*Sex*	*Age*	*AML subtype*	*Post-remission (days)*	*Cytogenetics*	*Molecular*	*Cyto risk*	*Relapse*	*Status*
130302	F	42	M4	168	45,XX,-7[16]/46,XX[4]		Poor	No	Deceased
130121	M	25	M1	259	46,XY[20]	NPM1 neg, FLT3 neg	Intermediate	No	Alive
130350	M	58	M5	189	46,XY,inv(12)(q13q24.3)[9]/46,XY[2]		Intermediate	Yes	Deceased
130473	F	42	M2	168	unsuscessful	NPM1 neg, FLT3 neg, AML1-ETO+, KIT neg	Not done	Yes	Lost to follow-up
130354	M	32	M4	216	inv(16) and t(16;16)	CBFB-MYH11+, KIT+	Favorable	No	Alive
130177	M	62	M4	399	46,XY,inv(16)(p13.1q22)[8]/46,XY[2]	CBFB-MYH11 +	Favorable	No	Alive
130429	F	67	M1	224	inconclusive	NPM1+, FLT3-TKD+	Not done	Yes	Alive
130873	F	60	M5	56	46,XX [20]	NPM1+, FLT3-TKD+	Intermediate	No	Alive
130363	F	23	Unclassified	189	45,X,Y,add(2)(p12),t(8;21)(q22;q22)[9]/46,XY[1]	C-kit+	Favorable	Yes	Deceased
130563	F	36	Unclassified	79	46,XX [20]	NPM1 neg; FLT3-ITD+ (interm.)	Intermediate	No	Alive
130574	F	43	M5	176	47,XX,+8,ins(9;11)(p22;q13q23)[12].ish ins(9;11)(p22;q13q23)(5'MLL+3'MLL+)/46,XX[2]	MLL rearrangement +	Intermediate	No	Deceased
130199	F	45	Unclassified	217	t(8;21)	C-kit neg	Favorable	No	Alive
130623	F	48	Unclassified	196	46,XX [20]	NPM1 neg, FLT3 neg	Intermediate	Yes	Alive
140079	F	32	Unclassified	47	46,XX,t(9;11)(q34;q23)[11]/46,XX[1]	MLL rearrangement +	Poor	No	Alive
130897	F	70	Unclassified	120	46,XX,del(2)(p11.2~21p13~23)[18]/46,XX[2].		Intermediate	Yes	Alive
130826	M	58	M4	161	46,XY[20]	NPM1 + FLT3-ITD+ (high)	Intermediate	Yes	Deceased
140012	F	56	M5	105	46,XX [20]	NPM1 + FLT3-ITD+ (interm.)	Intermediate	No	Deceased
140084	F	27	M3	91	t(15;17)	PML/RARA +	Favorable	No	Alive
130249	M	55	Unclassified	152	46,XY[20]	NPM1 + FLT3-ITD+ (low)	Intermediate	No	Alive
130346	F	76	Unclassified	221	46,XX [20]		intermediate	No	Alive

Abbreviations: ADCC, antibody-dependent cell cytotoxicity; AML, acute myeloid leukemia; F, female; M, male.
